# Research on Application of Japanese Quince (*Chaenomeles* L.) and Pork Collagen in Dark Chocolate—Benefits in Prevention of Inflammation In Vitro Model

**DOI:** 10.3390/nu16111758

**Published:** 2024-06-04

**Authors:** Szymon Byczkiewicz, Dominik Szwajgier, Ewa Baranowska-Wójcik, Aleksandra Telichowska, Krystyna Szymandera-Buszka, Janusz Wojtczak, Joanna Kobus-Cisowska

**Affiliations:** 1 Faculty of Food Science and Nutrition, Poznań University of Life Sciences, Wojska Polskiego 28, 60-637 Poznań, Poland; 58185@student.up.poznan.pl (S.B.); krystyna.szymandera_buszka@up.poznan.pl (K.S.-B.); 2 Department of Biotechnology, Microbiology and Human Nutrition, Faculty of Food Science and Biotechnology, University of Life Sciences in Lublin, Skromna 8, 20-704 Lublin, Poland; dominik.szwajgier@up.lublin.pl (D.S.); ewa.baranowska@up.lublin.pl (E.B.-W.); 3 Foundation for Education of Innovation and Implementation of Modern Technologies, 62-069 Dąbrówka, Poland; aleksandra.telichowska@gmail.com; 4 Department of Animal Breeding and Product Quality Assessment, Faculty of Veterinary Medicine and Animal Science, Poznań University of Life Sciences, Wojska Polskiego 28, 60-637 Poznan, Poland; janusz.wojtczak@up.poznan.pl

**Keywords:** chocolate, collagen, quince, antioxidant activity, functional food, enzymes

## Abstract

In the present study, the effect of the addition of quince and collagen type I and III to dessert chocolate on its functional properties was determined. The study evaluated the antioxidant potential of the tested formulations using the FRAP method and the linoleic acid oxidation test and beta-carotene bleaching test. The tested samples were also evaluated for inhibitory activity against enzymes important in preventive health (inflammation and neurodegenerative disorders) namely: AChE, BChE, GR, GPx, COX, and SOD. The addition of quince and collagen to the chocolate samples resulted in higher activity compared to the control sample, as indicated by the FRAP test. The experiment highlighted the impact of including quince fruit on the antioxidant activity of the chocolate samples. Interestingly, merely increasing the quince fruit amount did not consistently enhance antioxidant potential. Specifically, chocolate samples with a lower proportion of quince fruit (2 g/100 g) exhibited greater antioxidant activity when supplemented with collagen I. Conversely, in samples with higher quince percentages (3 g and 4 g), those enriched with collagen III showed higher antioxidant activity. Similar correlations were observed in the linoleic acid oxidation test. Notably, samples containing 3 g and 4 g of quince and type III collagen demonstrated statistically similar highest antioxidant properties. Regardless of the collagen type used, there was no observed increase in activity towards the tested enzymes for samples with the lowest percentage of quince fruit. Both collagen types exhibited the highest activity in the inhibition assay against acetylcholinesterase and butyrylcholinesterase when combined with 3 g and 4 g of quince. Overall, the experimental incorporation of both fruit and collagen enhanced the chocolates’ activity. Similarly to the antioxidant activity findings, chocolates with lower quince fruit quantities showed increased activity when supplemented with collagen III, while those with higher quince content (3 g and 4 g) displayed higher activity with collagen I. Bitter chocolate by itself is an attractive food product, rich in many bioactive compounds. However, enriching it with other attractive raw materials can make its properties and taste even more attractive.

## 1. Introduction

Chocolate is crafted from cocoa beans, typically in the form of cocoa pulp, along with cocoa fat and sugar. Available in various formulations, chocolates may contain different proportions of cocoa pulp and may feature additional ingredients such as flavorings, nuts, or fruits. The essential components, cocoa pulp and cocoa fat, are extracted from the seeds of the cocoa tree (*Theobroma cacao* L.), originating from Central and South America [1]. The main components of cocoa butter are significant amounts of fatty acids, including saturated (palmitic about 25%, stearic about 35%) and unsaturated (oleic about 38%, linoleic about 2%). In addition, free fatty acids (about 1.8%), antioxidant substances, minerals (mainly magnesium) and vitamins (of the B group, PP, A, E) are present. The functional properties of dessert chocolate are determined by numerous compounds with antioxidant properties including flavanols, among them epicatechin, epigallocatechin, or catechin. Cocoa also contains purine alkaloids—mainly theobromine (up to 1.8%). In addition to theobromine, caffeine is also present in small amounts [2]. 

Quince (*Chaenomeles* L.), a member of the rose family, originates from East Asia and is primarily grown in China. Renowned for its functional properties, this plant contains a diverse array of compounds including terpenoids, phenols, flavonoids, phenylpropanoids, benzoic acid derivatives, biphenyls, fatty acids, alkaloids, and numerous other compounds. In fact, around 203 chemical constituents have been isolated and identified within its composition. [3]. The literature data report that the plant has anti-inflammatory, anticancer, analgesic, antioxidant, hepatoprotective, neuroprotective, antibacterial, and antiviral properties. Quince is also a good source of vitamin C. Vitamin C affects the bioavailability of collagen. It plays an important role in the stabilization and synthesis of collagen fibers and is the most important component in the synthesis of hydroxyproline from proline [4]. 

Collagen constitutes roughly 30% of the total protein in animal bodies. The skeletal muscles of pigs and adult cattle contain between 1% to 10% collagen within their total protein composition. Approximately 25% of this protein is found in bones and blood vessel walls, while about 20% resides in the skin of both cattle and pigs. Collagen, regardless of its origin, is made up of 19 amino acids, including a key role played by hydroxyproline, which is not found in other proteins. The unusual amino acid composition of collagen is due to the high content of the amino acids glycine and proline, while cysteine, cystine, and tryptophan are absent (except for collagen III) [5,6]. 

Generally, collagen from higher vertebrates tends to be more cross-linked, leading to a higher denaturation temperature. During aging in higher animals, intramolecular and intermolecular cross-linking covalent bonds are produced in collagen, which cause the stability of collagen fibers. Thus, with an increase in the number of these bonds, collagen density and strength increase, while collagen solubility decreases [7].

As of now, researchers have identified and categorized 29 types of collagen. These types vary mainly in their structure, but also in their properties and distribution within the body [8]. Most studies focus on fibrillar collagen—in this regard, collagen types I, II, III, V, XI, XXIV, and XXVII are distinguished—and it accounts for about 90% of the collagen mass in the human body. This collagen is characterized by a structure of three helically coiled polypeptide chains containing specific fragments (composed of more than a thousand amino acid residues) located in the middle part of the chains [9]. 

This study used collagen types I and III, which are fibrillar collagen. They are found, respectively, in the skin, bones, ligaments, tendons, and cornea—collagen I—and in the skin, blood vessels, uterus, and intestine—collagen III. 

There is a current trend related to the production and popularity of functional foods. Given the popularity of dietary supplements with collagen, it was decided to develop a new functional snack in the form of dessert chocolate with added collagen. To increase the bioavailability of collagen, the addition of freeze-dried Japanese quince fruit, which is a good source of vitamin C, was included in the recipe. Vitamin C influences collagen synthesis by acting as a cofactor for enzymes responsible for the hydroxylation of proline and lysine in collagen precursors. These enzymes are prolyl hydroxylase and lysyl hydroxylase, which are necessary for proper formation of the collagen helix. Additionally, vitamin C contributes to the stabilization of collagen structure by participating in the formation of cross-links between collagen molecules. These cross-links provide collagen with strength and elasticity and act as an antioxidant, protecting collagen from damage caused by free radicals [9]. The aim of the study was to evaluate the functionality of the new snack in terms of assessing antioxidant activity and inhibitory activity against selected enzymes important in preventive health (inflammation). 

## 2. Materials and Methods

### 2.1. Materials

#### 2.1.1. Dark Chocolate Mass

The dark chocolate samples were acquired from the semi-industrial confectionery factory “BARS”, owned by Halina Kalemba, located in Wloszakowice, Poland. These samples, containing collagen types I and III with lyophilized Japanese quince were sweetened with polyols. The composition information and confirmed data about nutraceutical value of used chocolate mass is presented in the Table 1 below.

#### 2.1.2. Japanese Quince Fruit

The materials used in the study were Japanese quince fruits sourced from the “Szynsad” Orchard Farm in Dąbrówka Nowa near Grójec (latitude 51°46′56.093″ N, longitude 20°42′43.068″ E). The raw material was fruit of the *Chaenomeles japonica* Cido Japanese quince variety. Fruits were obtained from plants that had been grown for 6 years. The plant density in the orchard was 3300 pcs/ha. After harvesting, the fruits were washed in distilled water and dried, then the seeds were removed from them and crushed into cubes measuring about 1 × 1 cm. The raw material thus prepared was frozen at temperature = −28 °C until lyophilization. Lyophilization was conducted using a CHRIST 1-4 LSC freeze dryer (Martin Christ Ge-friertrocknungsanlagen GmbH, Osterode am Harz, Germany) under consistent conditions. The condensation temperature inside the freeze dryer was approximately −28 °C, with the shelf temperature set at 20 °C and the product temperature maintained at −4 °C. The entire process was conducted under reduced pressure for 24 h. Subsequently, the fruits were ground in a Grindomix GM 200 (Retsch, Haan, Germany) for 180 s at 1700× *g* and 21 °C.

#### 2.1.3. Collagen Preparate

Pork collagen preparation was obtained from a local distributor. The preparation was described as follows: Collagen type I—powder, light yellow color, neutral in taste and odor, solubility in water, protein content 92% in dry mass, dry substances 95%, minerals max 2.0%, pH 5.2. Microbiological data: total bacteria count < 1000 g, *E. coli*—negative, anaerobic sulfite-reducing bacteria < 10 g, *Salmonella* negative/25 g.

Collagen type III—powder, neutral in taste and odor, excellent solubility in water, protein content 97% in dry mass, dry substances 93%, minerals max 0.8%, pH 5.8. Microbiological data: total bacteria count < 1000 g, *E. coli*—negative, anaerobic sulfite-reducing bacteria < 10 g, *Salmonella* negative/25 g.

#### 2.1.4. Preparation of Chocolate Samples with Collagen and Japanese Quince Fruit

The chocolate was dissolved in a stainless-steel water jacketed heater equipped with a 100 rpm stirrer at 70 °C. Freeze-dried Japanese quince fruit was added in three concentration variants: 2 g, 3 g, and 4 g together with the collagen preparation by dissolving the mentioned additives initially in 1/4 of the intended volume of chocolate, and then in the entire chocolate provided by the recipe. The whole mixture was heated and stirred for 5 min, then poured into molds measuring 1 cm × 1 cm × 0.4 cm and left to cool at 21 °C. The cooled samples were stored until analysis at room temperature in tightly closed, dark containers.

The Table 2 below presents the composition of chocolate samples with collagen and Japanese quince fruit.

### 2.2. Chemicals

The following chemicals were obtained from Sigma-Aldrich (St. Louis, MO, USA): DMSO (D4540), AChE (C3389), BChE (C7512), ATChI (A5751), BTCh (B3128), DTNB (D8130), donepezil (D6821), neostigmine (N2001), eserine (E8375), CuCl2 (307483), TPTZ (93285), Trolox (238813), linoleic acid (L1376), Tween20 (P1379), β-carotene (C9750), Tween 80 (P1754), ascorbic acid (A92902), glutathione reductase (G3664), GSH (G4251), glutathione peroxidase (G6137), NADPH (N5130), EDTA (E9884), GSSG (G4626), SOD (S5395), nitrobluetrazolium (N6639), xanthine (X0626), and xanthine oxidase (X4875). AAPH was obtained from Acros Organics, Ottawa, ON, Canada (401560250), while COX-2 (human recombinant, 60122) and COX-2 activity assay kit (760151) were acquired from Cayman, Ann Arbor, MI, USA. Buffer salts, solvents, and other reagents were purchased from Sigma Aldrich (St. Louis, MO, USA) and were of at least analytical grade.

### 2.3. Methods

#### 2.3.1. FRAP Method

A sample volume of 0.02 mL was combined with 1.9 mL of FRAP solution and agitated for 30 min at room temperature. The absorbance was subsequently measured at 593 nm. The FRAP solution was prepared by blending 2.5 mL of 5 mM TPTZ solution (prepared in 40 mM HCl solution), 2.5 mL of 5 mM FeCl3 solution, and 25 mL of acetate buffer (0.3 M, pH 3.6), then warmed in a water bath at 37 °C for 20 min. A blank sample, containing only the reagent, was prepared using buffer in place of the sample, and the background of the sample was measured (a mixture containing the studied sample and buffer only). A calibration curve was established using 0.51 mg of Trolox dissolved in 1 mL of de-ionized water (DDI) (with the stock solution subsequently diluted to obtain 20 standard solutions in the range of 0.0255 to 0.51 mg Trolox per milliliter) [10]. 

#### 2.3.2. Linoleic Acid Oxidation Test

Linoleic acid (800 mg) was freshly dissolved in 20 mL of absolute MeOH and combined with 200 mL of 0.2 M sodium phosphate buffer (pH 6.5), resulting in a concentration of 6.5 mM Tween 20. The emulsion was sonicated for 10 min. Subsequently, 1.8 mL of the linoleic acid emulsion was mixed with 0.2 mL of the sample and then incubated at 37 °C. After 4 h of incubation, samples (0.1 mL) were extracted and mixed with 1.2 mL of absolute MeOH. The absorbance was measured at 234 nm against a blank sample lacking the studied solution. For the calibration curve, ten ascorbic acid solutions (ranging from 223.5 to 1676.3 μg ascorbic acid/mL) were prepared in the same manner as the test samples. The background of the sample was measured at 234 nm (a mixture containing the studied sample and buffer only) [11]. 

#### 2.3.3. Beta-Carotene Bleaching Test

β-Carotene (7 mg) was dissolved in 5 mL of chloroform along with 350 μL of linoleic acid and 2.8 g of Tween 80. The chloroform was evaporated under vacuum at 40 °C, and then 100 mL of deionized water saturated with oxygen was added and vigorously shaken. A 200 μL sample was combined with an equal volume of the β-carotene/linoleic acid emulsion. The absorbance at zero time and the change in absorbance were measured at 463 nm after 4 h at 50 °C. A stock solution of ascorbic acid (0.94 mg/mL) was prepared, and serial dilutions (9.4–94 μg/mL) were made and used instead of the studied samples as described above. The background of samples was measured (a mixture containing the studied sample and deionized water only). The percentage activity of samples was calculated based on the blank samples containing the emulsion and deionized water only [12]. 

#### 2.3.4. Effect on Cholinesterase (AChE and BChE) Activity

Ellman’s colorimetric method was used (Ellman et al., 1961) [13] with modifications described previously by Szwajgier and Baranowska-Wójcik 2019 [14]. The procedure involved mixing 1 μL of the tested sample with 20 μL of AChE (or BChE) solution (0.28 U/mL), followed by addition of 35 μL of ATChI (or BTCh) (1.5 mmol/L), 175 μL of 0.3 mmol/L DTNB (containing 10 mmol/L NaCl and 2 mmol/L MgCl_2_), and 110 μL of Tris-HCl buffer (50 mmol/L, pH 8.0) after a 5 min incubation. “Blank” samples, containing 35 μL of Tris-HCl buffer instead of the studied sample, were prepared in the same manner. The increase in absorbance due to the spontaneous hydrolysis of the substrate was monitored using “blank” samples containing ATCh (or BTCh) and DTNB, with the volume adjusted to 345 μL with Tris-HCl buffer. After incubation at 22 °C for 30 min (determined after optimization experiments), absorbance was measured at 405 nm using a 96-well microplate reader (Tecan Sunrise, Grödig, Austria). The “false-positive” effect of the studied compounds was assessed following the method of Rhee et al. 2003 [15] with minor modifications, as previously described by Szwajgier and Bar-anowska-Wójcik 2019 [14]: after mixing the substrate with the enzyme and buffer, the “false-positive” sample was incubated. Subsequently, a studied sample and DTNB were added, followed by an immediate measurement of absorbance. Reference cholinesterase inhibitors (eserine) were used for result calculations. For this purpose, 16 dilutions of each compound in pure DMSO were prepared (ranging from 2.57 to 41.14 μg/mL). These solutions (10 μL) were analyzed as described above, and calibration curves were generated. Each sample was analyzed in at least eight replicates, and all solutions used in a set of analyses were prepared in the same buffer. Background absorbance of the sample (10 μL mixed with 365 μL of Tris buffer) was measured at 405 nm and subtracted during calculations. Subsequently, the absorbance of the test sample was subtracted from the absorbance of the “blank” sample.

#### 2.3.5. Effect on Glutathione Reductase (GR) Activity

A volume of 0.02 mL of the sample was combined with 10 μL of EDTA solution and 12 μL of GSSG solution and incubated for 5 min at 25 °C. Subsequently, 4 μL of NADPH solution (all reagents dissolved in 0.1 mM sodium phosphate buffer, pH 7.6) was added, and the initial absorbance was recorded at 340 nm. The reaction was initiated by adding 2 U of glutathione reductase (2 μL, Sigma Aldrich no G3664) and 177 μL of 0.1 mM sodium phosphate buffer, followed by recording the absorbance after 5 min of incubation at 25 °C. The concentrations of reagents in the final mixture (805 μL) were as follows: 0.5 mM EDTA, 10 mM GSSG, and 10 mM NADPH. A blank sample was prepared with buffer instead of the sample, and the background was measured (a mixture containing the studied sample and buffer only). Enzyme activity was expressed as one unit, defined as nmol of NADPH consumed per minute per milliliter of sample, in comparison with the nmol of NADPH consumed per minute in the blank (reagent) sample [16].

#### 2.3.6. Effect on Glutathione Peroxidase (GPx) Activity

A volume of 0.020 mL of the sample was combined with 8 μL of EDTA solution, 10 μL of glutathione reductase (0.2 U, Sigma G3664), 4 μL of GSH solution, 10 μL of glutathione peroxidase (0.04 U, Sigma G6137), 22 μL of H_2_O_2_, and 332 μL of 50 mM sodium phosphate buffer (pH 7.0). To initiate the reaction, 4 μL of NADPH solution (N5130) was added, and the decrease in absorbance at 340 nm was measured after 10 min of incubation at 25 °C. All solutions were prepared in 50 mM buffer, and concentrations of reagents in the final mixture were as follows: 1 mM EDTA, 0.2 U glutathione reductase, 2 mM GSH, 0.04 U glutathione peroxidase, 1.5 mM H_2_O_2_, and 0.8 mM NADPH. A blank sample was prepared with buffer instead of the sample, and the background was measured (a mixture containing the studied sample and buffer only). Enzyme activity was expressed as one unit, defined as nmol of NADPH consumed per minute per milliliter of sample, in comparison with the nmol of NADPH consumed per minute in the blank (reagent) sample [17].

#### 2.3.7. Effect on Cyclooxygenase-2 (COX-2) Activity

For the assay, reagents from the Cayman COX-2 Assay Kit were prepared according to the manufacturer’s instructions and combined with COX-2 enzyme (Human recombinant, Cayman No. 60122, pre-diluted 100-fold using 100 mM Tris buffer, pH 8.0). A volume of 0.01 mL of the studied sample was mixed with 0.12 mL of Tris buffer (100 mM, pH 8.0), 0.01 mL of hemin, shaken, and left for 5 min at 25 °C. This was followed by the addition of 0.02 mL of the colorimetric substrate and 0.02 mL of arachidonic acid solution. The reaction was initiated by adding 0.02 mL of the COX-2 solution. The increase in absorbance during incubation at room temperature was recorded at 590 nm. Negative (blank) samples (buffer instead of the studied sample) and positive samples (COX-2 inhibitor DuP-697) were run simultaneously. The background of the studied samples (0.04 mL of sample mixed with 0.19 mL buffer) was also measured and included in the calculations. Each sample was run in at least 4 replicates. Inhibition of enzyme activity was expressed as a percentage (indicating the reduction in activity compared to the negative blank sample, which was assumed to have maximum activity of 100%, under the conditions used in the method). Additionally, inhibition of enzyme activity was expressed as acetylsalicylic acid equivalent concentration (mg/cm^3^). For this purpose, acetylsalicylic acid solutions were prepared at 14 concentrations (ranging from 0.2 to 10 mg/cm^3^) and analyzed similarly to the tested samples.

#### 2.3.8. Effect on Superoxide Dismutase (SOD) Activity

A volume of 0.05 mL of the sample was combined with 10 μL of SOD (0.24 U), 160 μL of nitrobluetetrazolium solution (0.0025 M), 205 μL of phosphate buffer (0.2 M, pH 7.5), 30 μL of xanthine (150 mM in 1 M NaOH), and 0.01 mL of xanthine oxidase (0.065 U, Sigma Aldrich X4875). The change in absorbance at 550 nm was measured in tested samples compared to controls without the studied sample after 20 min of incubation. The effect on the enzyme was calculated using the equation: Inhibition (%) = 100 − 100 × (Abs. 30 min − Abs. 0 min)/(Abs. control 30 min − Abs. control 0 min) [18].

### 2.4. Statistical Analysis

The routine statistical analyses included calculation of mean values and standard deviations. Statistical differences were determined using Tukey’s HSD test, with significance set at *p* < 0.05 (Statistica Software ver. 13.1, StatSoft, Krakow, Poland).

## 3. Results

Both chocolate and Japanese quince fruit contain natural antioxidants belonging to polyphenols, among others. Phenolic compounds are characterized by multidirectional effects. The enrichment of chocolate with Japanese quince fruit can help alter antioxidant potential and influence mechanisms that are crucial to maintaining health (against inflammation and neurodegenerative disorders). Regardless of their origin, polyphenols have the distinctive characteristic of being easily incorporated into redox reactions. The transfer of protons and electrons causes their oxidation, and the resulting quinones mediate the oxidation of other compounds that do not react directly with oxygen. 

In this study, in order to confirm the antioxidant activity of chocolate enriched with Japanese quince fruit and collagen, three spectrophotometric methods were used, including one post-additive FRAP and two methods in which the antioxidant potential of chocolate extracts was evaluated in fatty systems: linoleic acid oxidation and β-carotene oxidation test.

### 3.1. Content of Compounds Reducing Iron Ions (FRAP) in Chocolate with the Addition of Collagen and Japanese Quince, Linoleic Acid Oxidation and β-Carotene Oxidation Test

It was found that the addition of quince fruit influenced the antioxidant activity of the tested chocolate samples tested in the experiment. It was shown that increasing the share of Japanese quince fruit did not always increase the antioxidant potential (Table 3). For chocolate samples with a lower percentage of Japanese quince fruit (2 g/100 g), higher antioxidant activity was observed for samples with the addition of collagen I. However, in samples with a higher percentage of quince, i.e., 3 and 4 g, higher antioxidant activity was observed for samples with the addition of collagen III. In the FRAP test, the addition of quince fruit increased the activity in sample C33 with the addition of 3 g of quince to 0.30 mg/mL, then high iron ion reduction activity was found in samples C21 and C41, which amounted to 0.28 mg/mL. In the linoleic acid oxidation test, similar correlations were obtained as in the FRAP test. The sample with 4 g and 3 g of Japanese quince and type III collagen showed statistically similar highest antioxidant properties of 2.44 and 2.34 mg/mL (*p* ≤ 0.05). In contrast, the lowest activity was shown for the C21 and C41 sample (1.74–1.80 mg/mL). In the last test conducted, the lowest addition of quince and the two tested collagens did not have a statistically significant effect on the activity in β-carotene oxidation test (*p* ≤ 0.05). A higher share of Japanese quince fruit and type III collagen had a statistically significant effect in the test. The highest activity was observed in C33 and C41–C44 samples with type III collagen than for samples with type I collagen. The sample with 3 g of quince and 3 g of type III collagen (C33) showed the highest values in the test with β-carotene (44.3 µg/mL).

### 3.2. Inhibition of Acetylcholinesterase (AChE) and Butyrylcholinesterase (BChE) in Eserine

It was observed that regardless of the type of collagen used, there was no increase in activity towards the tested enzymes for the sample with the lowest percentage of quince fruit. In both the inhibition assay against acetylcholinesterase and butyrylcholinesterase, the highest activity was demonstrated with 3 g and 4 g quince and both collagens (Table 4). Moreover, in all cases, higher activity of the tested combinations of functional additives including collagen and quince was found in the test with BChE than with BChA.

Increasing the share of Japanese quince in the chocolate sample resulted in an increase in BChe activity regardless of the type of collagen—1.9–2.3 (μg/mL). Increasing the share of quince from 2 to 3 and 4 g increased the activity against AChE by 100%, and this activity was 0.7–0.8 (μg/mL).

### 3.3. Inhibition of Glutathione Reductase and Glutathione Peroxidase

Glutathione peroxidase (GPx) is an antioxidant enzyme and one of the markers of oxidative stress and inflammation disorders with selenium-dependent activity in the body, while glutathione reductase (GR) is a catalytic enzyme, working with all glutathione enzymes to reduce CSSG to GSH. The use of fruit and collagen in the production of experimental functional chocolates increased the activity in the tested experiment (Table 5). It was found, similarly to the case of antioxidant activity, that the samples with the lowest amount of quince fruit added showed higher activity with the addition of collagen III, while the samples with the higher amount of Japanese quince (3 and 4 g) showed higher activity with collagen I. The highest inhibitory activity against glutathione reductase (GR) was demonstrated by sample C21 and C43 with the addition of 2 g and 4 g of quince and type I and III collagen (57.8% and 55.4%), and the lowest by control sample CS (43.4%). For glutathione peroxidase (GPx), there was a similar tendency. 

### 3.4. Inhibition of Cyclooxygenase-2 and Superoxide Dismutase

Cyclooxygenase-2 (COX) enzyme is one of two forms of the enzyme cyclooxygenase-2. It is essential for physiological processes such as wound and ulcer healing. It is activated under the influence of factors associated with inflammation.

The cyclooxygenase-2 (COX-2) enzyme was inhibited by all chocolate samples with quince fruit and both collagen types (Table 6). All experimental tests were characterized by higher activity than a control test. There were no statistically significant differences in activity between functional tests with quince fruit and collagen (*p* ≤ 0.05). 

Superoxide dismutase (SOD) is an enzyme naturally found in the body, primarily in liver cells. It is one of the key compounds in the body’s defense against free radicals. In another study, it was checked which sample would best inhibit the superoxide dismutase (SOD) enzyme. It was found that the highest inhibitory activity in this regard was the C21 sample at 30.5%, and the lowest activity was shown by the C31 sample at 26.5%. All experimental samples were characterized by higher activity than the control sample. It was observed that samples with a lower share of fruit with the addition of collagen I showed higher activity, while for chocolates with 3 and 4 g of fruit, the activity was higher in the case of the simultaneous addition of collagen III.

## 4. Discussion

Chocolate is the most popular sweet treat in the world. Manufacturers seeking to increase its sensory and nutritional appeal use various types of functional additives. Not only nuts, but also fruits, their extracts, as well as probiotics (*L. rhamnosus*, *B. breve*, *B. animalis* subsp. *lactis*, *S. boulardii*, or *B. coagulans*) are added to chocolate. Combined with Japanese quince fruit and collagen, this product can be classified as a typical functional product. The enrichment of dessert chocolate with collagen is a novelty. Collagen has become a popular functional additive primarily in beverages and bars. 

In the present study, Japanese quince fruit, a good source of polyphenols and vitamin C with high antioxidant activity, was used in the composition of chocolate to affect the bioavailability of collagen. The study showed that the addition of quince fruit also affected the antioxidant potential of the chocolate. The FRAP test showed that the chocolate sample with Japanese quince and collagen addition had higher activity than the control sample. Researchers from Serbia indicated that the addition of fruit can affect the antioxidant properties of the product. They used a 4% addition of raspberries in dark chocolate and found a significant increase in antioxidant potential compared to a control sample without added fruit [19]. In our work, the addition of Japanese quince was chosen earlier, among hybrid varieties (Maksim, Gold Kalif and Cido)—the highest antioxidant potential by the FRAP method was indicated for Maksim (403 ± 32 (µM FeSO_4_/g dm.) [20]. 

In a paper published in 2023, researchers from Serbia evaluated the inhibitory capacity of dark chocolate enriched with the herbs *Salvia lavandulifolia* L., *salvia officinalis* L., and acerola fruit against the enzyme acetylcholinesterase (AChE). The results showed that as the content of polyphenolic compounds and enrichment of chocolate with additives increased, AChE inhibitory activity also increased. AChE inhibitory activity (IC_50_), ranged from 54 to 58 mg mL ^−1^ [21]. In the present study, in an inhibitory assay against acetylcholinesterase, the highest activity was shown in samples with 3 and 4 g quince and both types of collagen.

Evaluation of the activity of different types of chocolate enriched with phytochemicals extracted from yellow tea against the enzyme butyrylcholinesterase (BChE) was assessed in Gramza-Michalowska et al. BChE values ranged from 0.095 to 0.480 µM eserine/g sm. Evaluation of the chocolates’ activity against BChE showed that, prior to storage, BChE was inhibited to the greatest extent by dark and milk chocolate with yellow tea and milk chocolate without additives, while white chocolate with and without additives inhibited to the lowest extent [22]. Tests conducted in our study showed that BChE activity was best inhibited by samples with 4 g of Japanese quince and type III collagen (BChE 2.3c ± 0.2 (C23)).

The study by Kocakulak et al. 2023 examined the effect of dark chocolate consumption on markers of oxidative stress in athletes. A statistically significant change in superoxide dismutase (SOD) enzyme values was found in the group consuming chocolate before training (*p* < 0.05). A change in glutathione peroxidase GPx parameters was also observed in this control group (*p* < 0.05) [23]. In observing the superoxide dismutase (SOD) enzyme in the present study, it was observed that samples with a lower share of fruit with the addition of collagen I showed higher activity, while for chocolates with 3 and 4 g of fruit, the activity was higher in the case of the addition of collagen III. In our study, glutathione peroxidase (GPx) was more active in the sample with fruits and collagen than in control sample. The study by Latif et al. 2016 evaluated whether chocolate consumption can improve the oxidant–antioxidant balance in medical students. They found that SOD and GPx enzymes showed non-significant differences after 2 weeks of chocolate consumption (*p* = 0.46, 0.19, 0.11, and 0.06) [24]. 

Bitter chocolate by itself is an attractive food product, rich in many bioactive compounds. However, enriching it with other attractive raw materials can make its properties and taste even more attractive.

## 5. Conclusions

The FRAP test showed that the chocolate sample with quince and collagen addition had higher activity than the control sample. The experiment revealed that including quince fruit affected the antioxidant activity of the chocolate samples. Interestingly, simply increasing the amount of Japanese quince fruit did not consistently enhance the antioxidant potential. Specifically, for chocolate samples containing a lower proportion of quince fruit (2 g/100 g), those supplemented with collagen I exhibited greater antioxidant activity. Conversely, in samples with higher quince percentages (3 g and 4 g), those augmented with collagen III showed higher antioxidant activity. In the linoleic acid oxidation test, similar correlations were obtained as in the FRAP test. The sample with 4 g and 3 g of quince and type III collagen showed statistically similar highest antioxidant properties. It was observed that regardless of the type of collagen used, there was no increase in activity towards the tested enzymes for the sample with the lowest percentage of quince fruit. In the inhibition assays against both acetylcholinesterase and butyrylcholinesterase, the highest activity was demonstrated with 3 g and 4 g quince and both collagen types. GR and GPx enzymes were better inhibited by samples with added type III collagen. The quince fruit content had a limited effect on the increase in activity of the samples, which depended on the amount and type of collagen. The sample with 2 g of quince and type III collagen showed the highest inhibitory activity against glutathione reductase (GR), while the sample with 4 g of Japanese quince and type I collagen was the most effective against glutathione peroxidase (GPx). As a result of the work, a new snack with increased antioxidant activity and inhibitory activity against selected health-relevant enzymes was designed. It is necessary to check in the future how the new snack will interact in in vitro systems.

## Figures and Tables

**Table 1:** Nutraceutical value of chocolate.

Energy	545 kcal
Fat, incl:	31 g
Saturated fatty acids:	19 g
Trans fatty acids:	0.1 g
Cholesterol:	8 mg
Carbohydrates, incl:	61 g
Sugars:	48 g
Fiber:	7 g
Sodium:	24 mg
Potassium:	559 mg
Caffeine:	43 mg
Proteins:	4.9 g
Iron:	8 mg
Calcium:	56 mg
Magnesium:	146 mg

**Table 2:** Composition characteristics of chocolate samples with Japanese quince fruit and collagen.

Sample of Chocolate	Japanese Quince Fruit (g/100 g)	Collagen (g/100 g)/ Type of Collagen
CS	0	0/-
C21	2	2/type I
C23	2	2/type III
C31	3	3/type I
C33	3	3/type III
C41	4	4/type I
C43	4	4/type III

**Table 3:** Content of compounds reducing iron ions (FRAP) in chocolate with the addition of collagen and Japanese quince, linoleic acid oxidation and β-carotene oxidation test.

Sample	FRAP Equivalent Trolox Concentration (mg/mL)	Linoleic Acid Oxidation Equivalent Ascorbic Acid Concentration (mg/mL)	β-Carotene Oxidation Equivalent Ascorbic Acid Concentration (µg/mL)
CS	0.21 ^a^ ± 0.01	1.78 ^a^ ± 0.11	23.2 ^a^ ± 1.3
C21	0.28 ^c^ ± 0.01	2.38 ^c^ ± 0.05	27.0 ^b^ ± 1.2
C23	0.20 ^a^ ± 0.02	1.80 ^a^ ± 0.14	25.4 ^a^ ± 1.7
C31	0.24 ^b^ ± 0.03	2.16 ^b^ ± 0.11	26.9 ^b^ ± 2.1
C33	0.30 ^c^ ± 0.04	2.44 ^c^ ± 0.10	44.3 ^c^ ± 2.8
C41	0.28 ^c^ ± 0.02	1.79 ^a^ ± 0.09	31.1 ^b,c^ ± 1.0
C43	0.29 ^c^ ± 0.01	2.34 ^c^ ± 0.07	38.9 ^c^ ± 3.1

Results are mean values of three determinations ± standard deviation. Values sharing the same letter in a column are not significantly different (*p* ≤ 0.05).

**Table 4:** Inhibition of acetylcholinesterase (AChE) and butyrylcholinesterase (BChE) in eserine.

Sample	AChE (Eserine) μg/mL	BChE (Eserine) μg/mL
CS	0.4 ^a^ ± 0.0	0.8 ^a^ ± 0.2
C21	0.4 ^a^ ± 0.0	1.1 ^a^ ± 0.2
C23	0.4 ^a^ ± 0.0	1.0 ^a^ ± 0.1
C31	0.7 ^b^ ± 0.0	1.4 ^b^ ± 0.1
C33	0.8 ^b^ ± 0.2	1.3 ^b^ ± 0.0
C41	0.7 ^b^ ± 0.1	1.9 ^c^ ± 0.2
C43	0.7 ^b^ ± 0.0	2.3 ^c^ ± 0.2

Results are mean values of three determinations ± standard deviation. Values sharing the same letter in a column are not significantly different (*p* ≤ 0.05).

**Table 5:** Inhibition of glutathione reductase and glutathione peroxidase.

Sample	GR Inhibition (%)	GR Inhibitory Activity (μmol Consumed NADPH/min Incubation)	GPx Inhibition (%)	GPx Inhibitory Activity (nmol Consumed NADPH/min Incubation)
CS	43.4 ^a^ ± 1.8	1.7 ^a^ ± 0.1	35.6 ^a^ ± 3.5	74.8 ^a^ ± 3.5
C21	57.8 ^c^ ± 1.8	2.3 ^b^ ± 0.2	44.2 ^c^ ± 2.4	92.8 ^c^ ± 4.2
C23	49.5 ^b^ ± 3.1	1.9 ^a^ ± 0.2	34.6 ^a^ ± 3.0	72.6 ^a^ ± 3.2
C31	45.6 ^b^± 3.2	1.8 ^a^ ± 0.2	38.9 ^a,b^ ± 2.2	81.7 ^b^ ± 3.6
C33	48.3 ^b^ ± 1.9	1.9 ^a^ ± 0.3	45.8 ^c^ ± 4.2	92.0 ^c^ ± 4.9
C41	49.0 ^b^ ± 2.2	1.9 ^a^ ± 0.1	42.1 ^b^ ± 3.1	88.1 ^b^ ± 5.6
C43	55.4 ^c^ ± 2.9	2.2 ^b^ ± 0.2	45.6 ^c^ ± 3.3	95.86 ^c^ ± 3.5

Results are mean values of three determinations ± standard deviation. Values sharing the same letter in a column are not significantly different (*p* ≤ 0.05).

**Table 6:** Inhibition of cyclooxygenase-2 and superoxide dismutase.

Sample	COX-2 Equivalent Acetylsalicylic Acid Concentration (mg/cm^3^)	COX-2 Inhibition (%)	SOD Enzyme Inhibition (%)
CS	3.15 ^a^ ± 0.4	64.1 ^a^ ± 3.5	24.4 ^a^ ± 3.9
C21	3.29 ^b^ ± 0.1	96.1 ^c^ ± 2.1	30.5 ^c^ ± 1.5
C23	3.26 ^b^ ± 0.1	92.3 ^b^ ± 4.2	26.9 ^b^ ± 2.6
C31	3.24 ^b^ ± 0.1	87.2 ^b^ ± 3.1	26.5 ^b^ ± 3.6
C33	3.27 ^b^ ± 0.2	94.9 ^c^ ± 5.4	34.6 ^c^ ± 3.7
C41	3.26 ^b^ ± 0.1	86.7 ^b^ ± 4.4	33.4 ^c^ ± 2.1
C43	3.32 ^b^ ± 0.3	92.1 ^b^ ± 2.0	35.7 ^c^ ± 1.5

Results are mean values of three determinations ± standard deviation. Values sharing the same columns in a line are not significantly different (*p* ≤ 0.05).

## Data Availability

The original contributions presented in the study are included in the article, further inquiries can be directed to the corresponding author.
